# High-Performance Method for Brain Tumor Feature Extraction in MRI Using Complex Network

**DOI:** 10.1155/2023/8843488

**Published:** 2023-09-21

**Authors:** Thanh Han Trong, Hinh Nguyen Van, Luu Vu Dang

**Affiliations:** ^1^School of Electronics and Telecommunications, Hanoi University of Science and Technology, Hanoi, Vietnam; ^2^Department of Science and Technology Management and International Cooperation, Posts and Telecommunications Institute of Technology, Hanoi, Vietnam; ^3^Bach Mai Hospital, Hanoi, Vietnam

## Abstract

**Objective:**

To localize and distinguish between benign and malignant tumors on MRI.

**Method:**

This work proposes a high-performance method for brain tumor feature extraction using a combination of complex network and U-Net architecture. And then, the common machine-learning algorithms are used to discriminate between benign and malignant tumors. *Experiments and Results*. The dataset of brain MRI of a total of 230 brain tumor patients in which 77 high-grade glioma patients and 153 low-grade glioma patients were processed. The results of classifying benign and malignant tumors achieved an accuracy of 99.84%.

**Conclusion:**

The high accuracy of experiment results demonstrates that the use of the complex network and U-Net architecture can significantly improve the accuracy of brain tumor classification. This method could potentially be useful for clinicians in aiding diagnosis and treatment planning for brain tumor patients.

## 1. Introduction

In general, both benign and malignant brain tumors are potentially life-threatening, and the number of people with brain tumors is also increasing, according to statistics in the study of Ostrom et al. [1]. Therefore, early detection is a prerequisite to prevent the formation and growth of tumors to the next stage. Common methods of diagnosing brain tumors include Diagnosis through physical tests, CT scan [2], MRI [3], angiography, skull X-rays, biopsy, etc.

The current detection and differentiation of benign and malignant tumors is mainly thanks to the experience of specialists through imaging tests, blood tests, and, most precisely, biopsy. However, the biopsy of brain tumors is complicated, dangerous, and has the potential to cause complications. Therefore, specialists need to predict the likelihood of tumor nature before prescribing a biopsy based on careful evaluation of brain images. To be able to accurately detect and differentiate these types of tumors, doctors need to undergo a long process of intensive study and experimentation. Lashkari [4] showed that many types of tumors can be detected and classified based on MRI with different methods. The problem here is that with more and more patients, the medical system must process a huge number of images and doctors can be overloaded. Meanwhile, the requirements are given that the time of detection and classification needs to be fast and accurate. This dilemma was partly solved when the current stage of artificial intelligence was developed and has made great leaps. Artificial intelligence will help doctors classify diseases based on MRIs quickly and accurately.

A complex network [5] is described as a network including nodes that have very complex properties. In order to form a complex network, a large amount of information is needed to fully describe the topology of all network elements. Therefore, complex networks are expected to have outstanding advantages over conventional networks. In real life, complex networks can be used to describe many different real-world networks, such as social networks, networks of neurons in the brain, biological networks, etc.

Most recent studies on segmented 2D MRI of brain tumors [6, 7] have only focused on the detection of brain tumors. The segmented brain tumor has not been determined to be benign or malignant. There are also some studies that have only focused on testing in which MRIs are likely to show brain tumors, which do not indicate the specific location of the brain tumor. The method proposed by this paper will solve the above problem. In this study, we inherited the method of segmenting MRI of the brain to detect the location of brain tumors using U-Net network architecture, a good image segmentation method today. In particular, the core of this study is to focus on using complex networks to extract features of brain tumors after segmentation. Specifically, the complex network is used to model the pixel association graph of the tumor region, and the feature extraction of this graph is also the tumor feature extraction. From there, using machine learning algorithms to classify the characteristics of each tumor and predict whether the tumor is benign or malignant.

This paper is structured as follows: an introduction to the studies related to complex networks and brain tumor differentiation is presented in Section 2.  Section 3 provides an overview of MRI and data sets, knowledge about U-Net networks, classifier machine learning algorithms, and complex networks. The experiment results will be presented and compared with the results of previous studies in Section 4. Conclusions and development directions are in Section 5.

## 2. Related Work

There have been many studies on the technique of classifying brain tumors in MRI after segmentation and using traditional classification algorithms such as support vector machine (SVM), artificial neural network, K-nearest neighbor (KNN), naive Bayes (NB), logistic regression (LR), and random forest (RF). There are some articles with technical methods for classifying brain tumor images after segmenting brain MRIs from 2020 up to now, which are summarized in Table 1. The classification methods mainly use the traditional classification method based on the characteristics of the tumor characteristics. However, the difference between the methods is that different feature extraction algorithms are used, the goal of finding the most efficient classification algorithms.

Popular feature extraction methods are divided into two basic methods: using CNN network architecture and not using CNN network architecture. The authors in studies [8–12] used traditional feature extraction methods, whereas studies [8–11] used benign and malignant tumor images as data. Alves et al. [12] used data on inflammatory and brain tumors. Sarkar et al. [8] used the most traditional feature extraction method, the genetic algorithm, which includes 13 features such as mean value, standard deviation value, entropy, higher order moments, energy, contrast, inverse difference moment, correlation, smoothness, uniformity, and so on, to extract features of benign and malignant tumors. Then, the features are used for classification by the SVM algorithm with the performance of specificity 100%, sensitivity 98%, and accuracy 98.30%. Ansari et al. [10] also proposed a gray-level co-occurrence matrix (GLCM) method for feature extraction but combined with discrete wavelet transform (DWT) to get 12 features, also using SVM classification algorithm with an accuracy of 98.91%. Both Li et al. [11] and Alves et al. [12] used a synthesis of three traditional feature extraction methods: genetic algorithm, GLCM, and DWT. Meanwhile, Alves et al. [12] used additional gray-level run length (GLRL) to extract 63 different features and select the five best-ranked features. After using the classification algorithms SVM, kNN, and RFs, this study gave the performance of specificity 83.70%, sensitivity 91.20%, and accuracy 82.70%. Kang et al. [13] did not use traditional feature extraction methods, instead using 13 pretrained convolutional neural networks (CNNs) models for feature extraction. After selecting the top three pretrained CNN models as the models with the highest performance, the classification algorithms SVM, kNN, NB, and RFs were used and gave the highest accuracy of 98.50%. This result was the same in both SVM and kNN algorithms.

Jena et al. [14] used traditional methods for feature extraction, such as genetic algorithms, GLCM, GLRL, and DWT. After that, this study used three classification algorithms: SVM, kNN, and RFs; the results gave the highest accuracy with the RFs algorithm of 97%. Nanmaran et al. [15] used a new method for feature extraction of brain MRI, discrete cosine transform, in which six features of MRI were extracted. In that study, two algorithms, SVM and kNN, were used and gave an accuracy of 96.8% and 91.75%, respectively. Susanto et al. [16] also used the traditional feature extraction method, GLCM and DWT, to extract 16 features and classified by SVM algorithm with an accuracy of 98.65%. Finally, in the most recent study, Aamir et al. [17] used a new feature extraction method called multiple deep neural networks, also using SVM classification algorithm, and achieved an accuracy of 98.98%.

In recent years, there have been studies that have used complex networks for image processing [18, 19]. However, there are few studies on the application of complex networks in medical image processing in general and the classification of benign or malignant brain tumors in particular.

## 3. Materials and Methods

### 3.1. MRI and Dataset

Distinguishing clinical tumor status based on MRI [20] is a common method that medicine is using. This is a method for highly accurate results, especially in brain-related diseases. The classification of brain tumors can also be assessed based on MRI. Brain tumors often absorb drugs. From there, doctors can base density, location, or rate of drug absorption to diagnose a tumor with a high probability of being benign or malignant. For example, with a tumor with drug absorption but with a rough or irregular border, the rate is high for malignancy, while for tumors with a steady rate of enhancement, it is usually a tumor with a high rate of benignity. In addition, in case of enhancement of the annular border, there are other possible cases: brain abscess (a single foci and this is a benign mass); metastatic tumor (metastasis will have many different foci between the white matter and gray matter and cause brain edema) or Toxoplasma (appears in HIV patients).

### 3.2. Tumor Segmentation Using U-Net

To be able to extract features of brain tumors, the first thing we need to do is to segment the tumor. In this study, we use U-Net network architecture to automatically segment brain tumors on MRI. U-Net network architecture created by Ronneberger et al. [21], it was originally designed for the segmentation of medical images. Therefore, to segment brain MRI with benign and malignant tumors, in this study, we have researched and developed a U-Net network suitable for our study. U-Net network consists of two components: encoder and decoder. These two components include convolution operations, max pooling to increase or decrease the image size, and a total of 23 convolutional layers in the U-Net network. Strengths of U-Net network architecture compared to other segmented network architectures are shown as follows:

- U-Net network architecture does not use any fully connected layer (this layer is the layer that synthesizes image features to make prediction results).
- U-Net uses the padding method, which makes it possible for the architecture to fully segment the image.

### 3.3. Features Extraction

#### 3.3.1. The Theory of Complex Network

A complex network is a concept to describe a network consisting of nodes and edges whose relationships between nodes are complex and difficult to predict. In this study, the input MRI will be modeled by an undirected complex network. The input image will be divided into squares with dimensions of 2 × 2, 4 × 4, or 8 × 8, respectively. Each of those squares will be considered a node in the network. These nodes are considered related to each other based on three parameters: entropy, intensity, and position. Two nodes are linked if the following set of three conditions are satisfied:

- Two nodes are adjacent (position).
- The difference in intensity is negligible.
- The difference in entropy is negligible.

*(1) Entropy Matrix*. The entropy of a small window is often approximately the same as that of another, so it usually carries less information about the image. Furthermore, if the window size is reduced, the computational complexity in the analysis will increase rapidly. In this study, the entropy parameter was defined by Shannon [18], and the entropy of a square is calculated by the formula:

(1) $$\begin{array}{c} e=-\sum_{k}p\left(k\right)\log p\left(k\right), \end{array}$$

where *e* is the number of windows information, *p*(*k*) is the probability of the intensity value at the *k*th level. The probability *p*(*k*) with a luminance range between 0 and 255, divided by cells with a cell size of 4, is calculated as follows:

(2) $$\begin{array}{c} p\left(k\right)=\sum_{i=1}^{a}\sum_{j=1}^{a}p_{ij}\left(k\right)\left\{I_{ij}\epsilon b_{k}\right\}, \end{array}$$

where *I*_*ij*_ is the pixel brightness value at row *i*, column *j*; *p*_*ij*_ is the probability that the intensity appears in the *b*_*k*_ pane, and *a* is the size of the window. In fact, natural images often have very small changes in intensity at adjacent pixels. The intensity scale is divided into intervals that allow the intensity variations within the window to be evaluated by the entropy value. As the order of the interval is increased, the entropy difference between adjacent edges decreases. A pair of nodes will not be connected if the difference in entropy is greater than a given threshold.

*(2) Intensity Matrix*. The intensity of a node is the intensity of the pixels in that node and is calculated by the following formula:

(3) $$\begin{array}{c} m=\frac{1}{a^{2}}\sum_{i=1}^{a}\sum_{j=1}^{a}I_{ij}, \end{array}$$

where *m* is the intensity of a cell. Similarly, then *I*_*ij*_ is the intensity of the row *i* column *j* pixel and is the size of the cell. Two nodes are called connected if the difference in intensity of 2 pixels is less than a predetermined threshold. The weight of the link edge between the nodes will be formed.

*(3) Position Matrix*. This study only approaches the change of entropy and intensity of its adjacent nodes. A node is said to be adjacent to another node if its Euclidean distance is less than radius *r*. To ensure the continuity of the contour, the radius should be $\sqrt{2}$ [22]. Thus, the network is characterized for a maximum link level position of a node of 8. Nodes in the image contour usually have fewer links; the minimum number of link edges in the corner of the image is 3. The position elements have the following values:

(4) $$\begin{array}{c} \begin{array}{c} w_{P_{ij}}=\left\{\begin{array}{c} 0,\text{otherwise}\\ 1,if\sqrt{{\left(x_{i}-x_{j}\right)}^{2}+{\left(y_{i}-y_{j}\right)}^{2}}\leq \sqrt{2} \end{array}\right. \end{array}, \end{array}$$

where (*x*_*i*_, *y*_*i*_) and (*x*_*j*_, *y*_*j*_) is the position of node *i* and node *j* on the image.

*(4) Joint Matrix*. The joint matrix is created from the above three matrices as follows:

(5) $$\begin{array}{c} w_{EIP_{ij}}=w_{E_{ij}}.w_{I_{ij}}.w_{P_{ij}}, \end{array}$$

where *w*_*E*_*ij*__.*w*_*I*_*ij*__.*w*_*P*_*ij*__ are the weights corresponding to entropy, intensity, and position. If the value of the Joint matrix obtained in row *i* column *j* is 1, it means that the connection between the nodes has been created. Two nodes are considered connected if their pixels are in the same object or on the same edge. In this network, nodes with a degree of 8 are feature nodes located in the object, and less than 8 will be nodes of five on the boundary dividing the objects.

*(5) Boundary*. Han et al. [23] used a complex network combined with Wavelet Transform to distinguish between tumors and inflammatory regions in the human brain based on MRI. In that paper, the authors proposed a definition of the borderline of tumors and areas of inflammation on MRI. However, that proposal did not seem to be broad enough, only covering in a narrow solution space. A Complex Network can be created based on many parameters such as mean path, network diameter, and intensity of node. However, in the application of segmentation to extract features of human brain MRI with tumors (benign or malignant), the degree of each node is a particularly important parameter.

**Figure 1:**
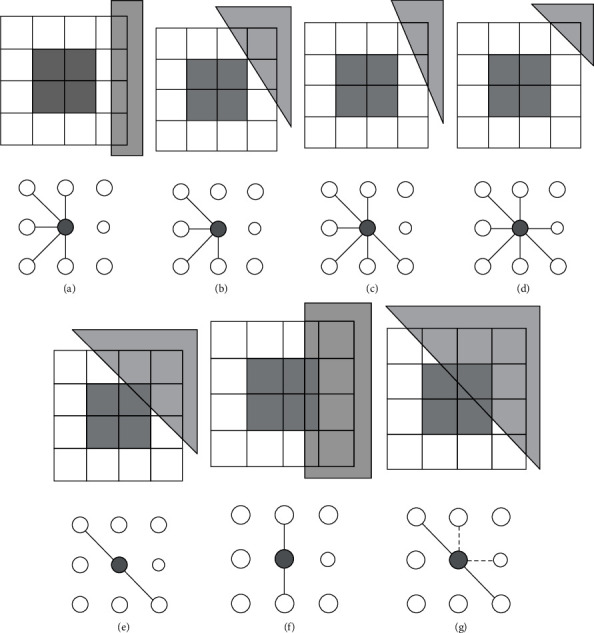
(a, b, c, d) The boundary passes through the combination of overlapping windows but not through the reviewed window. (e, f, g) The boundary passes through the combination of overlapping windows and through the reviewed window.

Obviously, when representing the image and determining the connection as above, a high-degree node will be in the center of the domain because there are many links with surrounding nodes. In other words, the central node is surrounded by windows similar to it. Conversely, nodes located on or near the boundary will have lower degrees. The degree parameter of each node in the network is inferred from the joint matrix that is set up above. In this paper, we propose the overlapping window method to scan the region and extract features of brain MRI. It is assumed that the window size is small enough that the traverse boundaries can be considered nearly straight.  Figure 1 shows a combination of eight overlapping windows surrounding a central window (reviewed window) corresponding to nine nodes. In which, each square window consists of four small squares.

When the boundary passes through a node (or window), the intensity and entropy of those nodes will be different from those inside the object. In case the boundary passes through the above combination but not through the center window, the center window will not be similar to at most three windows, so it will have a minimum degree parameter of 5 (as in Figure 1(a)–1(d)). Where the border passes through the center window along any line illustrated by Figure 1(e)–1(g), the smaller cases that can occur are as follows:

- In Figure 1(e), the boundary line passes through 1 quadrant of the viewing window (or it can be said that the area of the white region is more than eight times larger than the gray area). The reviewed window will only be similar to two corner windows (the area of the white area is also more than eight times that of the gray area), so the degree parameter of the reviewed window is 2.
- In Figure 1(f), the boundary line passes through the two quadrants of the reviewed window. The reviewed window will only be similar to the upper and lower windows (due to the large difference in the area ratio between the two regions between the reviewed window and the three right windows), so the window degree parameter is 2.
- In Figure 1(g), the boundary passes through three quadrants of the reviewed window. In addition to the similarity between the reviewed window and the two corner windows, the upper and right windows can also be similar to the reviewed window. Because the difference between the two domains and the reviewing window is not large, the existence of the link will depend on the threshold of the difference in entropy and intensity as well as the specific crossing position of the boundary. So, the reviewed window will have a degree parameter of 2 or 4.

#### 3.3.2. Feature Extraction Method Based on Pixel Graph Modeling Using Complex Network Theory

Feature extraction starts from an initially processed raw data set and builds values (features) from that data set to provide enough information and avoid information redundancy. This facilitates generalization and subsequent learning steps. And in some cases, it even helps people better interpret the problem. Object extraction is related to data size reduction. When the input data to an algorithm is too large to be processed and it is suspected to be redundant, it can be converted to subsets that reduce the size of the original objects (also named the initialization vector).

In the previous study of Lima et al. [24] on image processing using complex networks, the authors analyzed the basic features of graphs such as degree, centrality, and communities. From these basic features of the graph, the image has been remodeled, thereby giving the basic features of the image. An image can be graphically described based on the color patterns, texture, and shape of the image. An undirected graph *G*=(*V*, *E*) consists of *V* being the set of nonempty vertices and *E* being the set of unordered pairs of different elements of *V* called edges or connections between two pixels *i* and *j*.

The features of the graph can be mentioned as follows:

- Mean degree: Degree of a given vertex *i* is the number of vertices connected to it (its neighboring vertices). The average degree (*∅*_*μ*_) is the sum of the number of edges of the graph divided by the number of vertices of the graph.
- Average minimum path: The average minimum path is the average of all the minimum paths of the network.
- Mean intensity: The central mean is a measurement that represents the mean of the central peaks (the peaks that matter to the minimum paths of the network).
- Entropy of subgraphs: Quantify the randomness of the subgraphs *G*′ generated in the graph *G*.

The essence in the problem of image processing and image feature extraction is based on the values of image pixels, so modeling the tumor area is the modeling of the collection of pixels in the tumor area. Based on this statement, the complex network theory analyzed above is used to plot the set of pixels in the tumor region.

Principal components analysis (PCA) is a statistical algorithm that uses orthogonal transformations to transform a set of data from a high-dimensional space into a new lower dimensional space in order to optimize the representation of data variability. When using PCA to analyze the above four features, to keep 99% of the information of the data set, three of them are selected, which are the mean degree, mean intensity, and entropy of subgraphs.

### 3.4. Machine-Learning Algorithms

#### 3.4.1. Classification Methods

After segmenting the tumors, complex networks were used to extract features of the tumors. Those features will be the input to apply the classification algorithms. In this study, five classification algorithms were used: SVM [25], RFs [26], LR [27], NB [28], and kNN [29]. All five classification algorithms are traditional and widely used classification algorithms. The common feature of these classification algorithms is that they include a target variable, which is predicted from a set of independent factors.

Using these sets of input variables, we create a function that can allow the outputs to match the predictions corresponding to the input results. In this paper, the input of the algorithms is the three features extracted from the complex network method; the objective is the classification algorithm with the task of synthesizing and giving the unique features of the image with benign tumors and images with malignant tumors.

#### 3.4.2. Accuracy and F1-Score

In order to evaluate the performance of the training model, a set of indicators is needed to evaluate. In this paper, malignant tumors are considered positive, and benign tumors are considered negative. The parameters true positive (TP), false positive (FP), true negative (TN), and false negative (FN) are defined in Table 2.

Accuracy is the ratio between the correct prediction score and the total score in the test data set. Precision is defined as the ratio of the number of true positives among those that are classified as positive. Recall is defined as the ratio of true positives to positives. F1-Score is the harmonized average between precision and recall.

Accuracy, precision, recall, and F1-score indicators in binary classification are expressed in the following formulas:

(6) $$\begin{array}{c} \text{Accuracy}=\left(\text{TP}+\text{TN}\right)/\left(\text{total sample}\right), \end{array}$$

(7) $$\begin{array}{c} \text{Precision}=\text{TP}/\left(\text{TP}+\text{FP}\right), \end{array}$$

(8) $$\begin{array}{c} \text{Recall}=\text{TP}/\left(\text{TP}+\text{FN}\right), \end{array}$$

(9) $$\begin{array}{c} F1=2\times \left(\text{precision}\times \text{recall}\right)/\left(\text{precision}+\text{recall}\right). \end{array}$$

In order to evaluate the effectiveness of the model in classification problems, the receiver operating characteristic (ROC) curve is often used. This is the parameter generated from the confusion matrix. For the multiparameter classification problem, For the multiparameter classification problem, area under the curve (AUC-ROC) is an important parameter used for evaluation. In ROC space, the curve plot is constructed based on false positive rate (FPR) and true positive rate (TPR). TPR is the ratio of the correct number of positive class predictions of the model to the total number of actual active classes. FPR is the ratio of the number of false predictions of the model's positive class to the total number of actual negative classes. The area under the ROC curve is called the AUC. The prediction accuracy of the models was evaluated based on this AUC parameter. The higher the AUC value, the more accurate the model is. The models are evaluated as good when AUC ≥ 0.8, reliable when 0.7 ≤ AUC < 0.8, and unreliable when AUC < 0.7.

## 4. Experiments and Results

### 4.1. Data Collection

**Figure 2:**
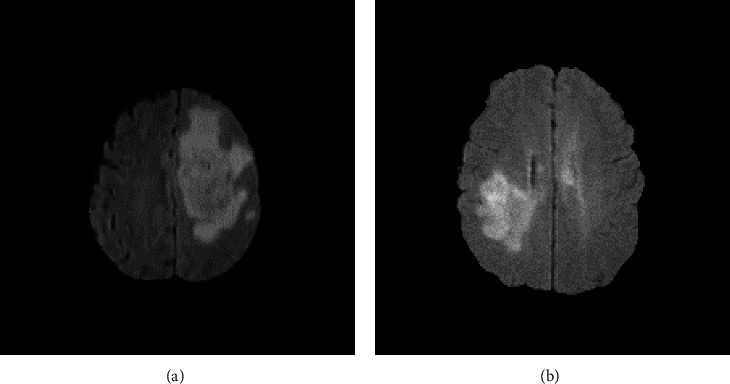
MRI with FLAIR sequence: (a) HGG; (b) LGG.

This dataset includes MRI of 230 brain tumor patients. This is an open dataset of MRI from the Center for Biomedical Image Computing & Analytics, Perelman School of Medicine, University of Pennsylvania [30–32]. This data set, after being collected, has been thoroughly checked for correctness and labeled by specialists at the radiology center, Bach Mai Hospital, Vietnam. The dataset includes 77 high-grade glioma (HGG) patients and 153 low-grade glioma (LGG) patients.  Figure 2 depicts MRI brain tumor images of HGG and LGG diseases.

### 4.2. Classification Process

The input of the image segmentation is a 3D MRI of the above dataset with two image modes: T2 and T2-FLAIR. The 3D MRI will be cropped to slides. These slides are 2D MRI; we will remove the 2D MRI that has no value for brain tumor image segmentation. The total number of 2D MRI for segmentation is 2,683 images, of which, T2 pulse sequence has 474 images representing HGG, 953 images representing LGG; T2-FLAIR pulse sequence has 425 images showing HGG, 831 images showing LGG.

**Figure 3:**
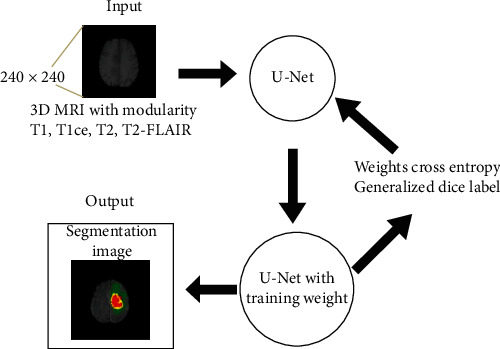
Brain tumor image segmentation process.

Then, the images will be fed into the U-Net model to train. The training process helps the trained weights get better. After the training is complete and the best set of weights is returned, the patient's brain tumor images will be included for image segmentation.  Figure 3 depicts the image segmentation process through the U-Net model.

**Figure 4:**
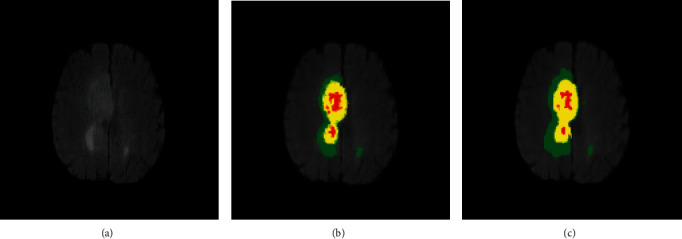
Brain segmentation results: (a) FLAIR image; (b) GT image; (c) segmentation image.

The model is trained with 60% images of the dataset. The image output shows that the image of HGG disease is more accurate than that of LGG disease, and the segmentation results on the whole tumor are more accurate on each region.  Figure 4 depicts the results of MRI of brain tumors after segmentation with red the tumor nucleus, yellow the necrotic area, and blue the inflammatory area.

**Figure 5:**
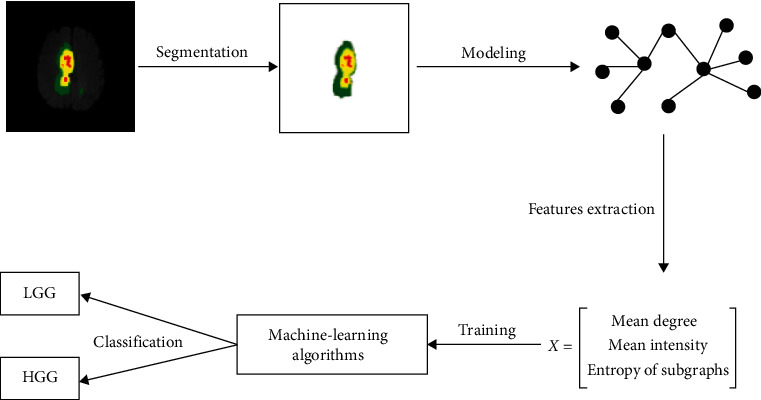
Tumor classification process after segmentation.

After segmenting, the tumor portion will be modeled as a graph by the complex network. From there, the three characteristics, mean degree, mean intensity, and entropy of subgraphs, will be extracted. This process is shown in Figure 5.

Then, the extracted features will be fed into five classification algorithms: SVM, RFs, LR, NB, and kNN. The data used in this step are divided according to the ratio 6:2:2 (60% for training, 20% for the training validation process (validation), and 20% for testing), corresponding to the number of training images is 1,609; the number of validation images is 536 images, and the number of images to test is 538 images.

### 4.3. Classification Results

#### 4.3.1. Experiment Results

**Figure 6:**
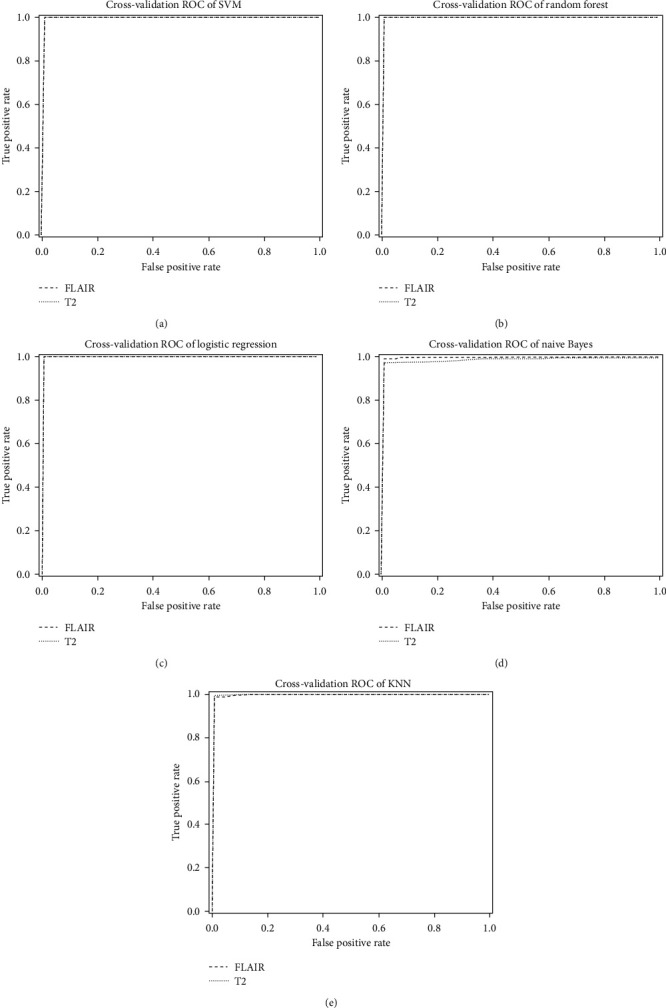
ROC curve on two types of MRI: T2 and T2-FLAIR of five algorithms: (a) SVM; (b) random forest; (c) logistic regression; (d) naive Bayes; (e) KNN.

In this study, we used three features extracted from tumor images after segmenting T2 and T2-FLAIR images as described above. The obtained results are presented in Figure 6 and Table 3. Figure 6 plots the results of the curve after training five classification algorithms, SVM, RFs, LR, NB, and kNN, with input data of three features above. The ROC curves have shown good classification results, all giving AUC > 0.95 results.

Table 3 presents the classification accuracy of five classification algorithms: SVM, RFs, LR, NB, and kNN. The classification algorithms all give results with an accuracy of over 96%. Comparing the image classification work in this study with the classification of medical images in general, this is a result with good reliability and can be applied in practice. Brain MRI with T2 pulses has the following results: the highest accuracy is 99.84%, the highest precision is 99.93%, the highest recall is 100%, and the highest F1-score is 99.96%. With the T2-FLAIR pulse brain MRI, the obtained results are as follows: The highest accuracy is 99.69%, the highest precision is 100%, the highest recall is 99.89%, and the highest F1-score is 99.80%.

When comparing the results between the classification algorithms, the LR classification algorithm gives the best accuracy result of 99.84%, followed by the RF, kNN, NB, and SVM algorithms with results of 99.71%, 98.84%, 98.55%, and 99.69%, respectively. Especially for the F1-score index, the classification algorithm gives the highest accuracy of 99.96% in the LR algorithm. It shows that the LR classification algorithm is the most suitable algorithm with the feature obtained from the proposed method.

#### 4.3.2. Comparison of Result

All the previous studies that we collected used the traditional classification algorithms SVM, RF, LR, NB, and kNN, so we compared the accuracy of the results obtained in this paper with the results of previous studies; the specific comparison results are presented in Table 4. Obviously, it is easy to see that the accuracy achieved in this paper for all classification methods is much higher than that achieved by previous studies.

Table 5 shows a comparison of the accuracy and number of features used between previous studies and this study. Most of the classification algorithms give high accuracy due to a large set of different classification features. However, the proposed method has overcome this drawback when only three features are needed, but the obtained accuracy is highest. Research by Kang et al. [13] also uses only three features. However, those three features are top-3 deep features (DenseNet-169 feature, ShuffleNet V2 feature, and MnasNet). They are drawn from 13 pretrained CNN models, where deep features are a collection of different small features. Therefore, with only using three basic features, the computational complexity in this study is likely to be improved over the computational complexity encountered by Kang et al. [13].

When considering the aspect of the application of complex networks, Han et al. [23] also used complex networks in combination with wavelet transform techniques to extract features of tumors and inflammatory regions in the human brain on MRI. In that study, the authors had to use four features, with three features taken from the complex network. Although not for the same purpose, it can be seen that in this paper, with the overlapping window method, the study only needs to use three features obtained from the complex network. With only those three features, basic machine learning algorithms such as SVM, RFs, LR, NB, and kNN have enough information to be able to classify benign and malignant tumors with high accuracy.

## 5. Conclusion

This paper proposed a method that can automatically classify brain tumors as benign or malignant. The core of the study is the proposal of a tumor feature extraction method using complex networks. Those features were obtained from segmented tumors using U-Net and then fed to machine learning methods for tumor classification. The obtained experimental results have proved the effectiveness of the proposed method with higher accuracy than previous studies.

Other previous studies needed a lot of features to represent the information of the MRI. Meanwhile, with the feature extraction method using complex networks, only three features are needed to show almost all the information of the MRI. Those three features have helped machine learning algorithms successfully classify benign or malignant tumors with the highest accuracy of 99.84%. Thus, the use of only three features has made the extraction quick and time-consuming but still highly efficient; this is the basis for applying the model in practice.

The field of image processing using complex networks is quite new, and this is also an opportunity and challenge for the research community. Our next research direction is to model image graphs using complex networks for different problems, not only the problem of classifying benign or malignant tumors but will also apply to the classification of the properties of each tumor type or other types of information.

## Figures and Tables

**Table 1:** Summary of machine learning-based brain tumor classification techniques.

Studies	Dataset	Feature extraction method	Number of features	Classifier	Tumor types	Performance
Sarkar et al. [8]	Local dataset: 60 MRI T2 images	Genetic algorithm	13 features	SVM	Benign, malignant	SPE 100%, SEN 98%, ACC 98.30%

Hamid et al. [9]	Local dataset: 60 MRI T2- FLAIR images	GLCM	5 features	SVM	Benign, malignant	ACC 95%

Ansari et al. [10]	Local dataset: 200 MRI	GLCM and DWT	12 features	SVM	Benign, malignant	ACC 98.91%

Li et al. [11]	Local dataset: 135 MRI T1 and T2 images	Gabor transform, texture, and DWT	80 best-ranked features	SVM	Benign, malignant	SPE 80%, SEN 93%, ACC 88%

Alves et al. [12]	Local dataset: 67 patients T1, T1C+, T2, DWI, T2-FLAIR	Genetic algorithm, GLCM, GLRL and DWT	Five best-ranked features	SVM kNN RF	Brain tumors and inflammatory lesions	SPE 83.70%, SEN 91.20%, ACC 82.70%

Kang et al. [13]	Local dataset: 6,517 MRI	CNN	Top-3 deep features	SVM kNN NB RF	Benign, malignant	SVM: ACC 98.50% kNN: ACC 98.50% NB: ACC 90.20% RF: ACC 97.17%

Jena et al. [14]	BraTS 2017 and 2019	Genetic algorithm, GLCM, GLRL and DWT	471 features	SVM kNN RF	LGG, HGG	SVM: ACC 94.25% kNN: ACC 87.88% RF: ACC 97%

Nanmaran et al. [15]	Local dataset: 200 MRI	Discrete cosine transform	6 features	SVM kNN	Benign, malignant	SVM: ACC 96.8% kNN: ACC 91.75%

Susanto et al. [16]	BraTS 2019	GLCM and DWT	16 features	SVM	LGG, HGG	SVM: ACC 98.65%

Aamir et al. [17]	Local dataset: 3,064 MRI	Multiple deep neural networks	Deep features	SVM	Benign, malignant, pituitary	SVM: ACC 98.98%

GLCM, gray-level co-occurrence matrix; LGG, low-grade glioma; HGG, high-grade glioma.

**Table 2:** Confusion matrix.

Actual	Predicted	
	Positive	Negative
Positive	TP	FN
Negative	FP	TN

**Table 3:** Results of evaluation parameters of five classification algorithms on T2 and T2-FLAIR MRI.

Type MRI	Classifier	Accuracy (%)	Precision (%)	Recall (%)	F1-score (%)
T2	SVM	99.38	99.23	100	99.61
	Random forest	99.71	99.91	99.72	99.81
	Logistic regression	99.84	99.93	99.98	99.96
	Naive Bayes	95.78	97.34	97.33	97.29
	kNN	98.84	99.56	98.98	99.26

T2-FLAIR	SVM	99.69	100	99.61	99.80
	Random forest	99.66	99.89	99.68	99.78
	Logistic regression	99.69	99.71	99.89	99.80
	Naive Bayes	98.55	99.60	98.58	99.07
	kNN	98.77	99.07	99.38	99.22

**Table 4:** Compare accuracy with several similar classifiers.

Classifier	Paper	High accuracy (%)
SVM	Sarkar et al. [8]	98.30
	Hamid et al. [9]	95.00
	Ansari et al. [10]	98.91
	Li et al. [11]	88.00
	Alves et al. [12]	76.60
	Kang et al. [13]	98.50
	Jena et al. [14]	94.25
	Nanmaran et al. [15]	96.8
	Susanto et al. [16]	98.65
	Aamir et al. [17]	98.98
	This study	99.69

kNN	Alves et al. [12]	80.60
	Kang et al. [13]	98.50
	Jena et al. [14]	87.88
	Nanmaran et al. [15]	91.75
	This study	98.84

Naive Bayes	Kang et al. [13]	90.20
	Jena et al. [14]	97
	This study	98.55

Random forests	Alves et al. [12]	82.70
	Kang et al. [13]	97.17
	This study	99.71

**Table 5:** Accuracy comparison.

Paper	Feature extraction method	Number of features	High accuracy (%)
Sarkar et al. [8]	Genetic algorithm	13 features	98.30
Hamid et al. [9]	GLCM	5 features	95.00
Ansari et al. [10]	GLCM and DWT	12 features	98.91
Li et al. [11]	Gabor transform, texture, and DWT	80 best-ranked features	88.00
Alves et al. [12]	Genetic algorithm, GLCM, GLRL, and DWT	Five best-ranked features	82.70
Kang et al. [13]	CNN	Top-3 deep features	98.50
Jena et al. [14]	Genetic algorithm, GLCM, GLRL, and DWT	471 features	97
Han et al. [22]	Complex network + wavelet transform	4 features	93.06
Nanmaran et al. [15]	Discrete cosine transform	6 features	96.8
Susanto et al. [16]	GLCM and DWT	16 features	98.65
Aamir et al. [17]	Multiple deep neural networks	Deep features	98.98
This study	Complex network	3 features	99.84

GLCM, gray-level co-occurrence matrix.

## Data Availability

The data used to support the findings of this study are available in the references.
